# Autogenous bone grafts in oral implantology—is it still a “gold standard”? A consecutive review of 279 patients with 456 clinical procedures

**DOI:** 10.1186/s40729-017-0084-4

**Published:** 2017-06-01

**Authors:** Andreas Sakkas, Frank Wilde, Marcus Heufelder, Karsten Winter, Alexander Schramm

**Affiliations:** 1 Department of Oral and Plastic Maxillofacial Surgery, Military Hospital Ulm, Academic Hospital of the University of Ulm, Oberer Eselsberg 40, 89081 Ulm, Germany; 2 Institute of Anatomy, Medical Faculty of Leipzig University, Leipzig, Germany; 3 Department of Oral and Plastic Maxillofacial Surgery, University Hospital Ulm, Ulm, Germany

**Keywords:** Autologous bone augmentation, Gold standard, Intraoral bone grafts, Complications, Dental implants, Donor site

## Abstract

**Background:**

This study assessed the clinical outcomes of graft success rate and early implant survival rate after preprosthetic alveolar ridge reconstruction with autologous bone grafts.

**Methods:**

A consecutive retrospective study was conducted on all patients who were treated at the military outpatient clinic of the Department of Oral and Plastic Maxillofacial Surgery at the military hospital in Ulm (Germany) in the years of 2009 until 2011 with autologous bone transplantation prior to secondary implant insertion. Intraoral donor sites (crista zygomatico-alveolaris, ramus mandible, symphysis mandible, and anterior sinus wall) and extraoral donor site (iliac crest) were used. A total of 279 patients underwent after a healing period of 3–5 months routinely computer tomography scans followed by virtual implant planning. The implants were inserted using guided oral implantation as described by Naziri et al. All records of all the consecutive patients were reviewed according to patient age, history of periodontitis, smoking status, jaw area and dental situation, augmentation method, intra- and postoperative surgical complications, and surgeon’s qualifications. Evaluated was the augmentation surgical outcome regarding bone graft loss and early implant loss postoperatively at the time of prosthodontic restauration as well a follow-up period of 2 years after loading.

**Results:**

A total of 279 patients underwent 456 autologous augmentation procedures in 546 edentulous areas. One hundred thirteen crista zygomatico-alveolaris grafts, 104 ramus mandible grafts, 11 symphysis grafts, 116 grafts from the anterior superior iliac crest, and 112 sinus lift augmentations with bone scrapes from the anterior facial wall had been performed. There was no drop out or loss of follow-up of any case that had been treated in our clinical center in this 3-year period. Four hundred thirty-six (95.6%) of the bone grafts healed successfully, and 20 grafts (4.4%) in 20 patients had been lost. Fourteen out of 20 patients with total graft failure were secondarily re-augmented, and six patients wished no further harvesting procedure. In the six patients, a partial graft resorption was detected at the time of implantation and additional simultaneous augmentation during implant insertion was necessary. No long-term nerve injury occurred. Five hundred twenty-five out of 546 initially planned implants in 259 patients could be inserted into successfully augmented areas, whereas 21 implants in 20 patients due to graft loss could not be inserted. A final rehabilitation as preplanned with dental implants was possible in 273 of the 279 patients. The early implant failure rate was 0.38% concerning two out of the 525 inserted implants which had to be removed before the prosthodontic restoration. Two implants after iliac crest augmentation were lost within a period of 2 years after loading, concerning a total implant survival rate after 2 years of occlusal loading rate of 99.6% after autologous bone augmentation prior to implant insertion.

**Conclusions:**

This review demonstrates the predictability of autologous bone material in alveolar ridge reconstructions prior to implant insertion, independent from donor and recipient site including even autologous bone chips for sinus elevation. Due to the low harvesting morbidity of autologous bone grafts, the clinical results of our study indicate that autologous bone grafts still remain the “gold standard” in alveolar ridge augmentation prior to oral implantation.

## Background

Oral implantation has a significant role in the rehabilitation of patients. Bone reconstruction techniques have been advanced in order to optimize the esthetic and functional outcome. However, the restoration of the oral function of atrophic alveolar crests still remains a challenge in oral implantology. Bone augmentation procedures are often indicated to allow implant placement in an optimal three-dimensional position to obtain long-term function and predictable esthetic outcome for prosthodontic restorations [1]. The extent of atrophy of the alveolar crest dictates whether the bone augmentation procedures may be performed simultaneously with the implant placement or as a separate procedure [2].

Among the different available augmentation materials, only autologous bone combines osteoconductive, osteoinductive, and osteogenic characteristics compared to bone substitute and composite materials [3]. Because of its properties and absence of immunological reactions, autologous bone grafts have been considered as the “gold standard” and most effective material in bone regeneration procedures [4–7]. Success rates exceeding 95% have been achieved, even when major augmentation procedures with autologous bone had to be carried out for severely resorbed jaws [8, 9]. However, limitations of autografts which include restricted donor sites and possible harvesting morbidity, reports of unpredictable resorption, and limited available bone volume had been reported for intraoral bone grafts [1, 9].

The most frequently applied bone augmentation techniques are staged guided bone regeneration procedures which include transplantation of an autologous bone block adding mechanical support to the covering soft tissues [10]. A number of different donor sites offering membranous or endochondral bone from regional or distant sites are available. The grafts differ considerably as far as embryology, histology, mechanical properties, and the volume that can be harvested are concerned. The choice of a specific donor site often is based on a number of different aspects like the expected donor site morbidity or bone resorption rate [8, 9]. Especially, sinus floor elevation is an established method of bone augmentation in the atrophic posterior maxilla [11].

Although the iliac crest is most often used in jaw reconstruction, a significant bone resorption has been mentioned [12]. This disadvantage, and the fact that dental implants do not always require a large amount of bone, has increased the use of autologous block bone grafts from intraoral sources [13]. Bone grafts from intraoral donor sites offer several benefits like surgical accessibility, proximity of donor and recipient sites, and less discomfort for the patient and less morbidity as compared with extraoral locations [14].

Intraoral harvesting has been reported to be associated with a relevant morbidity, and the significant graft resorption or their oral exposures are two of the most frequently reported complications [15]. The choice of the intraoral donor site is usually based on the amount, geometry, and type of bone required for alveolar reconstruction, and additionally, the incidence of intra- and postoperative complications should be considered [16, 17]. Six systematic literature reviews related to lateral atrophic ridges regenerated with intraoral bone block grafts have been reported and found evidence of bone gain and high implant success rates [18–22].

In the recent years, several alternatives have been investigated to supply the reported disadvantages of autologous bone. Allogenic grafts have been extensively used obtained from individuals from the same species but with different genetic load [23]. An allogenic graft is considered to be biocompatible with great applicability, exhibits good postoperative response without donor site morbidity, and is available in unlimited quantities [24, 25]. Furthermore, the anorganic bovine bone has received attention in the literature, since it yielded a long-term success in ridge augmentation technique. It is widely used for vertical and horizontal augmentation, sinus lift procedures, and socket treatment after tooth extraction [26]. While for sinus lift procedures bone substitutes have proven to achieve reliable results, we still lack clinical evidence that bone substitutes are equally reliable for horizontal and vertical augmentation of the dentoalveolar process.

In our military outpatient center exclusively, autologous bone transplantations harvested from different donor sites were used intraorally (crista zygomatico-alveolaris, ramus mandible, symphysis mandible, anterior sinus wall) and extraorally (iliac crest) to reconstruct severe horizontal and/or vertical alveolar ridge atrophy prior to implant placement. The aim of this study was to assess the clinical outcomes in terms of postoperative complications and harvesting morbidity, graft success rate, and implant survival rate, in a 2-year follow-up after alveolar ridge reconstructions with autologous bone grafts.

## Methods

### Patient selection

For this retrospective cohort study, we reviewed the records of all patients without exclusion criteria who were referred to the department of oral and plastic maxillofacial surgery at the military hospital of Ulm, Germany, between January 2009 and December 2011 for alveolar ridge augmentations prior to implant insertions using autologous bone grafts harvested from different donor sites and unilateral or bilateral sinus floor elevations with a lateral approach. There was no dropout or loss of follow-up of any patient that had been treated in our clinical center in this 3-year period (dropout rate = 0).

The files of 279 patients with a total of 456 augmentation procedures were reviewed.

At the first appointment for all included 279 patients, the medical and dental history as well as smoking habits was recorded using a standardized questionnaire.

During the initial clinical examination, the periodontal status was determined by means of a comprehensive periodontal assessment: lost teeth, teeth with bone loss over 5 mm, mobility grade, and periodontal pocket >4 mm were used to define a history of periodontal disease. Approximal Plaque Index (API) according to Lange and Sulcus Bleeding Index (SBI) according to Mühlemann and Son were also assessed at the first clinical examination [27, 28].

Occlusal and prosthodontic analyses were performed clinically as well as with the aid of dental casts.

The need for restorative treatment was also stated during this first appointment.

The preoperative radiological assessment included careful evaluation of the dental status and any pathologic conditions using panoramic X-rays and, potentially, computed tomography.

### Indication for bone augmentation

Indication for the need for bone augmentation procedures was determined by means of the following parameters:

- Presence of severe alveolar ridge atrophy rated classes IV and V according to the Cawood and Howell classification [29]
- Residual maxillary bone less than 5 mm from the alveolar crest to the sinus floor

The indication for augmentation of the alveolar ridge defect was evaluated on the basis of a clinical examination with oral inspection and the use of dental casts and a radiological examination using panoramic radiographs to observe the height and width of the alveolar ridge and to identify structures of risk like the mandibular canal or the maxillary sinus. Three-dimensional radiographs had been used when clinical examination and two-dimensional radiographs were not sufficient to prove alveolar ridge dimensions. In 30.8% (83/279 patients), preoperative 3D CT examination had to be performed to assess the need and volume of bony reconstruction needed prior to implant insertion. All patients were informed in advance that bone grafting was necessary prior to implant placement because of the inadequate bone quality. Occlusal analysis was performed, diagnostic wax-ups were prepared on the articulated casts, and restorative treatment needs to be determined.

### Surgical protocol

#### Bone block onlay graft procedures

A standardized two-stage surgical protocol was used, and all sites were treated in a similar fashion. In the first intervention, a bone block harvested from the donor site was fixed with osteosynthesis titanium screws to the recipient site as an onlay graft to achieve a horizontal and/or vertical enlargement of the alveolar ridge. Placement of the bone graft was always guided by an augmentation template as described by Schramm et al. [30–32]. In the second procedure, 3 to 5 months later, the screws were removed and the implants were placed using guided oral implantation as described by Naziri et al. The number of bone blocks and donor sites and the number of implants inserted in each augmented site were recorded. The choice of donor site, either left or right, was determined preoperatively based on defect morphology and recipient site location. Every bone harvesting procedure was performed using the same standardized surgical technique.

Intraoral autologous bone block grafts were harvested using piezoelectric surgery from the following donor sites:

- Lateral zygomatic buttress (crista zygomatico-alveolaris)
- Ramus mandible in the retromolar area
- Symphysis mandible

An oscillating saw and/or chisels were used for harvesting bone from the inner surface of the iliac crest (crista iliaca anterior superior).

The recipient site was dissected and pre-conditioned using a Safescraper device (C.G.M. S.p.A., Divisione Medicale META, Italy). The collected bone was preserved in a sterile environment until grafting. The block grafts were fixed with the aid of 1.0–2.0-mm diameter titanium osteosynthesis screws, and bone chips were packed around the bone blocks to fill gaps between the bone blocks and recipient buccal/labial wall. Any rough edges on the bone blocks were smoothed with a rotating burr and diamond burr. The entire graft was always covered by a collagen membrane (Bio-Gide, Geistlich Biomaterials, Wolhusen, Switzerland) and the periosteum was released to ensure a tension-free closure. The flap was closed with 3/0, 4/0, and 5/0 resorbable sutures.

Grafting from the iliac crest was always performed under general anesthesia in a two-team approach. The iliac crest was exposed and autogenous grafts from the anterosuperior inner edge of the iliac wing were harvested with an oscillating saw and/or a chisel, keeping a safe distance of around 2 cm from the anterosuperior iliac spine. After harvesting the bone grafts, the corticocancellous bone blocks were positioned by means of the technique described above.

The quantity of the bone needed for augmentation from the different donor sites was always depended on the size and form of the alveolar ridge defect and was evaluated always clinically before harvesting. The exact dimensional measurement of the bone blocks harvested and their radiological comparison in order to assess the resorption rate at the time of implantation was not investigated in this study. Each donor site provided a different bone quality according to the form and thickness.

#### Sinus lift procedures

Unilateral or bilateral sinus floor elevations in a two-stage procedure were also included in this study. The sinus augmentations were carried out with autogenous bone chips from the lateral sinus wall gained with a scraper device (C.G.M. S.p.A., Divisione Medicale META, Italy) as well as from the iliac crest, when the operation was combined with onlay grafts from the iliac crest.

Where the sinus lift procedures were concerned, the incision was made on the top of the alveolar ridge cutting the keratinized attached mucosa. A mucoperiosteal flap was raised, and the preparation started with the bone scraper (Safescraper; C.G.M. S.p.A., Divisione Medicale META, Italy). The bone from the anterior and lateral walls of the sinus was collected as part of the antrostomy. The preparation was concluded with the aid of a large round diamond bur to minimize the risk of Schneiderian membrane perforation. The Schneiderian membrane was carefully elevated using special mucosal sinus elevators until sufficient space for the impaction of bone material was created.

In addition to the bone already gained with the bone scraper device from the sinus wall during the antrostomy, bone was harvested with the same device from the maxillary buccal buttress, if more volume was needed. By taking this approach, the collection of enough bone for the augmentation of at least two implantation sites was feasible with a mean surgical time of 5 to 10 min for harvesting. In cases where an additional augmentation was performed with grafts from the iliac crest, the sinus lifting was performed with spongeous bone chips from the iliac crest instead of the bone gained locally with the bone scraper.

After impaction of the bone graft material in the sinus cavity, the bony sinus window was covered with a resorbable collagen membrane (Bio-Gide®, Geistlich Biomaterials, Baden-Baden, Germany). Finally, the mucoperiosteal flap was replaced and sutured without periosteal release if no additional bone block augmentation was performed at the same operation site.

#### Implant placement

After a healing period of 3 to 5 months, computed tomography scans were performed, followed by virtual implant planning using coDiagnostiX^®^ software (Dental Wings GmbH, Chemnitz, Germany). After transfer of the planning into surgical guides, the augmented regions were re-opened, the screws which had been inserted during bone block augmentations were removed, and the implants were inserted using guided oral implantation as described by Naziri et al. [33]. All patients were recalled every 6 months for clinical and radiological examination within a period of 2 years after prosthodontic rehabilitation.

The implant success rate was clinically and radiographically evaluated over a follow-up period of 2 years after prosthesis loading according to the criteria of Buser et al. described below [34]:

- Implant in situ
- No permanent disorders such as pain and dysesthesia
- No peri-implant infection
- No implant mobility
- No persistent peri-apical radio-translucency

### Data collection

The following data were collected from the patients’ medical files regarding bone augmentation during the postoperative healing period until prosthetical rehabilitation:

- Medical history of patient
- Age of patient at the time of bone harvesting and augmentation
- History of periodontal disease
- Smoking habits
- Donor site
- Jaw area and dental situation of the recipient site
- Intraoperative complications
- Postoperative complications after augmentation
- Management of complications
- Bone graft stability and clinical resorption prior to implant placement
- Complications after implantation in a 2-year follow-up after prosthesis
- Experience of surgeon (resident or consultant)

### Complications

Complications related to autologous bone augmentation and implant procedures were registered using the following definitions:

- Intraoperative complications
  - Intraoperative perforation of the Schneiderian membrane
- Early postoperative complications
  - Soft tissue dehiscency, when a separation of the suture line with or without exposure of the barrier membrane occurred
  - Wound infection/inflammation characterized by pain, swelling, redness, fever and/or purulent discharge that required additional antibiotic treatment
  - Bone graft exposure with or without screw mobilization
  - Sensory disturbance if altered sensation at the neural supply area of alveolar inferior nerve, lingual nerve, and infraorbital nerve was recorded after surgery
  - Symptoms of sinusitis after surgery on the posterior maxilla
  - Secondary hemorrhage at the donor or recipient site
- Late postoperative complications
  - Surgical removal of the bone graft, defined as *bone graft failure*
  - Early implant loss, when assessed before the placement of prosthetical restorations
  - Late implant loss, when assessed within 2 years after prosthetical restorations
  - Sensory disturbance, if altered sensation at the neural supply area of alveolar inferior nerve, lingual nerve, and infraorbital nerve was recorded at the time of reentry for dental implantation 4 to 5 months after bone augmentation.

Additional augmentation procedures with bone chips needed at the time of implant placement to obtain sufficient implant coverage as a result of partial graft resorption or inadequate primary augmentation were recorded. The dimensions of the bony defects and the quantitative success of the bone augmentation were not measured in this study. Due to the retrospective design of this study and the use of resorbable membranes and since all implants were inserted submerged, no reentry procedure was indicated to allow clinical evaluation of the augmented hard tissue volume. When re-augmentation procedures were needed by patients who suffered from a bone graft failure, the second procedures were not included in our statistical evaluation.

### Classification of implant failure

Early and late implant loss was documented in this study, defining the clinical success of osseointegration. Early implant failures were assessed before the acquisition of osseointegration, i.e., before the placement of prosthodontic restorations. Early implant failure could occur from the time of placement, during the healing phase and before abutment connection. The implant inserted after re-augmentation was not included in the survival rate analysis. Late implant failures were documented within a period of up to 2 years after loading of the prosthodontic restorations. Implant and prosthodontic restoration design were not evaluated.

### Result presentation

Demographic data and complication rates were presented descriptively. The results were additionally analyzed in percentage terms and presented in the form of tables and diagrams.

## Results

### Patient characteristics

Two hundred seventy-nine patients—250 men and 29 women—underwent 456 augmentation procedures involving autologous bone grafts prior to implant placement. The patients ranged in age from 18.5 to 71.5 years (average 43.1 years) at the moment of augmentation surgery.

Of those patients, 162 (58.1%) were younger than 40 years of age and 117 (41.9%) were older than 40 years of age. Caries or periodontitis was, in the majority of cases, the cause of primary tooth loss (89.9%, *n* = 251), followed by trauma in 20 (7.1%) of the cases, and agenesis in eight cases (3.0%).

Regarding the alveolar crest situation preoperatively, 163 defects were recorded as single-tooth gap, 119 as free-ending dental arch, and 79 as tooth gap involving more than one tooth. Edentulism was observed in 19 of the cases. Three hundred one bone harvesting procedures were performed for augmentation of the maxilla and 155 for the mandible.

One hundred ninety of 279 patients were operated in the maxilla, while 69 patients were augmented only in the maxilla, and 20 patients were treated in the maxilla and mandible.

Ninety-three (33.3%) of the patients were smokers and 186 were non-smokers.

With reference to the gingival/periodontal indices, 174 (62.3%) patients had an API score <20%, while 105 (37.7%) patients had an API score ≥20 and ≤30% prior to augmentation. The SBI showed a score of <20% in 213 (76.3%) patients and ≥20 and ≤30% in 66 (23.7%) patients. Patients with higher scores than 30% had not been treated since this was the prerequisite to implant treatment. As regards of SBI, 213 (76.3%) patients showed a score <20%, in contrast to 66 (23.7%) patients with SBI score 20–30%.

Patient characteristics are listed in Table 1.

**Table 1:** Patient characteristics at the time of augmentation

Patient characteristics	*N* (%)
Gender^a^	
Male	250 (89.6%)
Female	29 (10.4%)
Age^a^	
<40 years	162 (58.0%)
>40 years	117 (42.0%)
Smoking habits^a^	
Smokers	93 (33.3%)
Non-smokers	186 (66.6%)
API score^a^	
<20%	174 (62.3%)
≥20%	105 (37.6%)
SBI score^a^	
<20%	213 (76.3%)
≥20%	66 (23.6%)
Cause of tooth loss^a^	
Caries/periodontitis	251 (89.9%)
Trauma	20 (7.1%)
Hypodonty	8 (3.0%)
Augmentation site in the upper or lower jaw^a^	
Only maxilla	190 (68.1%)
Only mandible	69 (33.9%)
Both maxilla and mandible	20 (7.1%)
Dental situation^b^	
Tooth gap	99 (21.7%)
Single-tooth gap	163 (35.7%
Free-end dental arch	154 (33.7%)
Edentulous	40 (8.9%
Surgeon^c^	
Residents	223 (35.2%)
Senior consultants	410 (64.8%)

^a^ *N* refers to the total number of the study patients (*N* = 279)

^b^ *N* refers to the total number of the augmented areas (*N* = 456)

^c^ *N* refers to the total number of the surgical approaches (*N* = 633)

Three hundred donor sites were necessary to perform the 456 augmentation procedures. One hundred thirteen bone grafts were harvested from the zygomatic buttress (crista zygomatico-alveolaris), 104 grafts from the mandibular ramus (retromolar area), 38 grafts from the iliac crest (spina iliaca anterior superior), 36 grafts from the lateral sinus wall, and 9 grafts from the mandibular symphysis.

A total of 112 sinus floor elevations were performed. In all of the cases, implants were inserted in a two-stage procedure. The donor site for harvesting the bone for the sinus elevations was in 76 procedures in the iliac crest area, and in 36 procedures, the bone was harvested with a bone scraper device from the lateral sinus wall at the site of sinus lifting.

The distribution and number of transplanted grafts are illustrated in Table 2 according to different donor sites and grafting methods.

**Table 2:** Donor sites and numbers of bone grafts as well as distribution in patients in this study

Donor site	Bone grafts (*N*)/patients (*N*)
Lateral zygomatic buttress	113/112
Mandibular ramus (retromolar)	104/86
Mandibular symphysis	11/9
Iliac crest for alveolar ridge augmentation Iliac crest for sinus floor elevation	116/38 76/38
Bone chips harvested with a Safescraper device from the lateral sinus wall for sinus floor elevation	36/34
Total	456/279

Where the surgical experience of the surgeons is concerned, residents performed 223 (35.2%) procedures in total, whereas consultants performed 410 (64.8%) procedures.

### Surgical outcome

Four hundred thirty-six of 456 augmentation procedures were performed successfully.

Seven of the 104 mandibular ramus grafts, two of the 113 zygomatic buttress grafts, and one of the 11 symphysis grafts were lost. Out of the 38 iliac crest procedures, there were eight regions with graft failures. In two sinus floor elevations after two-stage procedure, bone grafting failed. Twenty bone grafts failed in total (4.3%).

Fourteen of 20 patients with total graft failure were augmented secondarily. Three patients with retromolar graft loss were re-augmented with contra-lateral retromolar grafts. Two patients with loss of zygomatic buttress grafts underwent re-augmentation after graft harvesting from the contra-lateral site. One patient who lost a mandibular symphysis graft was re-augmented with a retromolar graft, and eight patients who lost their iliac crest grafts at the implant site were also re-augmented with bone blocks from the mandibular ramus. The healing period for all the re-augmented patients was uneventful, and implants could be inserted without any further complications after integration of the bone grafts.

Six of the 20 patients who suffered from a graft loss (four after retromolar bone grafts and two after sinus floor elevation) wished no further augmentation procedure and were treated with a conventional prosthetic restoration.

In six patients, a partial graft resorption was detected at the time of implantation and an additional simultaneous augmentation with bone chips harvested with the Safescraper device (C.G.M. S.p.A., Divisione Medicale META, Italy) was then necessary in order to ensure the osseointegration of the implants. Two out of these six cases had grafts from the crista zygomatico-alveolaris, two from the ramus mandible and two from the iliac crest.

**Fig. 1:**
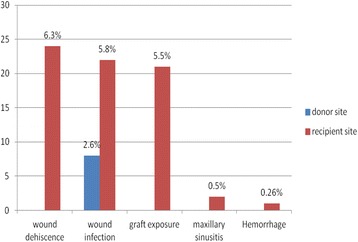
Postoperative complications at the donor and recipient site, *N* refers to the total number of the donor sites (*N* = 300), *N* refers to the total number of the recipient sites (*N* = 378)

**Fig. 2:**
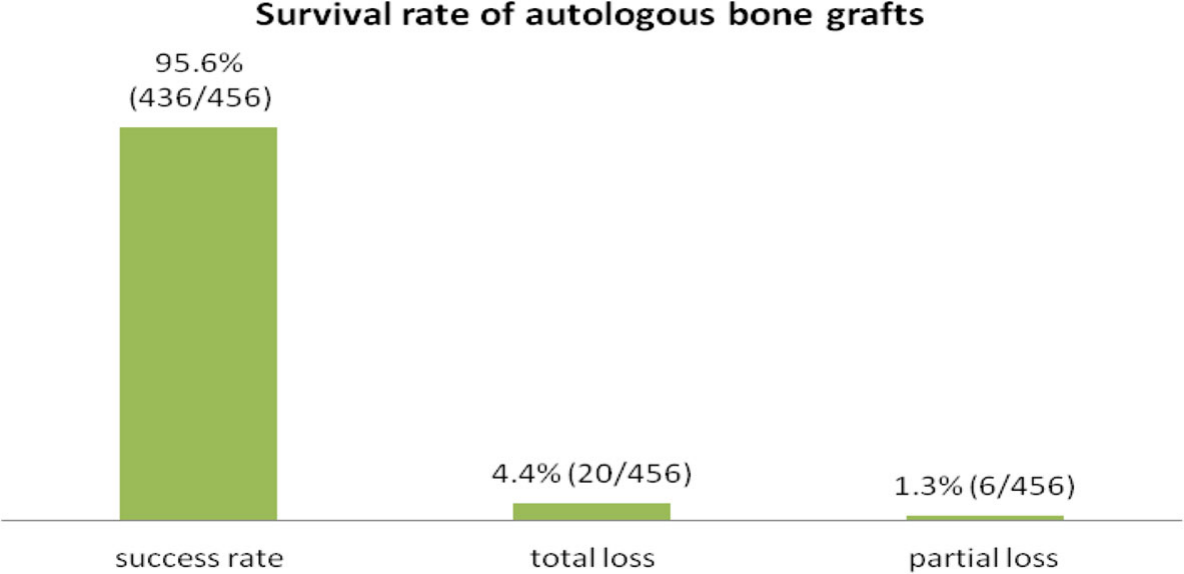
Survival rate of autologous bone grafts

**Fig. 3:**
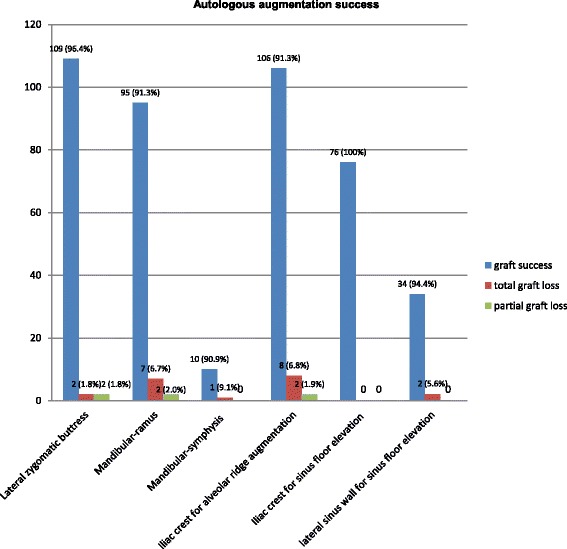
Surgical outcome after autologous augmentation procedures from different donor sites

Distribution of the harvesting methods, intra- and postoperative complications of the donor and recipient site, and bone graft survival related to the different autologous augmentation procedures are listed in Table 3 and presented in Figs. 1, 2, and 3.

**Table 3:** Intra- and postoperative complications after autologous bone harvesting

Postoperative complications	%/procedures (*N*)
At donor site^a^	
Wound infection	2.6% (8/300)
At recipient site^b^	
Soft tissue dehiscence	6.3% (24/378)
Wound infection	5.8% (22/378)
Graft exposure	5.5% (21/378)
Maxillary sinusitis	0.5% (2/378)
Hemorrhage	0.26% (1/378)
Nerve disturbance	
Temporary hypoesthesia mental nerve after grafting from ramus mandible	10.5% (11/104)
Temporary hypoesthesia lingual nerve after grafting from ramus mandible	2.8% (3/104)
Temporary hypoesthesia mental/lingual nerve after grafting from symphysis mandible	0%
Temporary hypoesthesia infraorbital nerve^c^	2.6% (6/225)
Sinus membrane perforation^c^	4.8% (11/225)

^a^ *N* refers to the total number of donor sites (*N* = 300)

^b^ *N* refers to the total number of recipient sites (*N* = 378)

^c^ *N* refers to the total number of the surgical approaches in the maxilla (*N* = 225)

### Crista zygomatico-alveolaris

One hundred thirteen zygomatic bone grafts were harvested in 112 patients. Two out of the 112 patients were treated in two different alveolar sites while the rest of the patients in only one atrophic area for augmentation of the maxilla. Of the 113 onlay bone graft procedures, 93 (82.3%) were defined as completely successful, while 20 (17.7%) had adverse effects, such as soft tissue dehiscence, swelling, wound infection, or graft exposure. Of the total areas with complications, four were defined in the donor site and 20 in the recipient area. Two patients developed postoperative symptoms of maxillary sinusitis in combination with persistent fistula at the donor site. By these two patients, a perforation of the maxillary sinus membrane was noted intraoperatively. The frequency of complications was higher in recipient sites. Except for minor complications such as soft tissue dehiscence (*n* = 8), wound infection and abscess formation were observed in five augmented areas and bone graft exposures in seven of the 113 cases. In two cases (1.7%), the bone graft was totally exposed in combination with wound infection and discharge of pus. The surgical removal of the graft was then inevitable; these two patients wished no further bone grafting operation and were finally treated with a conventional bridge reconstruction. Totally, 134 implants had been inserted in 111 augmented sites. In two cases, two implants were inserted with simultaneous bone chip augmentation because of partial bone graft resorption. None of the inserted implants did fail due to lack of osseointegration at the time of prosthodontic restoration.

### Ramus mandible (retromolar)

A total of 104 retromolar bone graft procedures in 86 patients were conducted. Twenty-two harvesting procedures were performed for augmentation of the maxilla and 82 for the mandible. Seven retromolar bone grafts (93.2%) in seven single-tooth gap dental regions by seven patients had been lost. Therefore, seven implants could not be inserted in augmented alveolar sites after graft failure. Three of the patients with total graft failure were secondarily augmented with retromolar grafts, harvested from the contra-lateral site, and all initially planned dental implants had been successfully inserted. The other four patients wished no further surgical procedure and were treated with a conventional prosthodontic restoration. A total of 155 implants had been inserted in 97 augmented sites. In 95 of the 97 cases, the implant insertions were uneventful; in two cases, the need of additional simultaneous bone chip augmentation due to partial graft resorption was essential. None of the inserted implants failed due to lack of osseointegration at the time of prosthodontic restoration.

### Symphysis mandible

Eleven bone graft procedures harvesting from the mandibular symphysis were performed in nine patients. All procedures involving the mandible were done in the same surgical field for donor and recipient site. Two out of nine patients were treated in two different alveolar sites, while the rest of the patients in only one atrophied area. Of the 11 onlay bone grafts, 10 (90.9%) were defined as completely successful. In one patient, wound infection and abscess formation were developed and the bone graft had to be surgically removed. This patient after wound healing was re-augmented with a bone graft from the ramus mandible and could be successfully restored with an implant prosthesis as initially planned. A total of 10 dental implants were inserted in 10 augmented sites. None of the inserted implants failed due to lack of osseointegration at the time of prosthodontic restoration.

### Iliac crest

Thirty-eight patients underwent a total of 116 augmentation procedures harvesting from the iliac crest. In 20 patients, a bone graft augmentation of the maxilla and the mandible in combination with bilateral sinus floor augmentations was performed. Eighteen patients had augmentations only in the maxilla, involving bone grafting and sinus lift elevations. Totally, 76 sinus lifts with bone material from the iliac crest had been performed. Eight grafts in eight patients developed wound infection combined with graft exposure, and the grafts were surgically removed. These patients were re-augmented with bone blocks from the mandibular ramus in the respective regions of bone loss. According to the donor site, one of 38 patients appeared with wound infection at the harvested iliac crest site. In the recipient sites occurred a higher complication rate. One case of soft tissue dehiscence, wound infection, and abscess formation was observed by six augmented areas and bone graft exposures in six of the cases. In addition, one patient showed a postoperative hemorrhage at the augmented area. A total of 187 implants had been inserted in 106 augmented areas. In two cases, two implants were inserted with simultaneous bone chip augmentation because of partial bone graft resorption. Two implants in two patients out of the 187 inserted implants were removed before the final prosthodontic restoration. One hundred eighty-five inserted implants showed regular osseointegration at the time of prosthodontic loading. Two implants were lost within 2 years after loading. These two implants concerned two patients after iliac crest graft augmentation and had to be removed due to development of peri-implantitis; the first 18 months and the second about 20 months after loading.

### Sinus floor elevation

One hundred twelve sinus floor elevations had been performed in 72 patients to treat severely atrophic posterior maxilla using autogenous bone grafts. In 34 patients (36 sinus lifts), a sinus elevation was performed with bone material from the lateral sinus wall, while 38 patients (76 sinus lifts) underwent bone harvesting from the iliac crest. Of the 112 sinus floor elevations, 95 (84.8%) were uneventful, and 17 (15.2%) had adverse effects, such as swelling, wound infection with discharge of pus, or acute symptoms of sinusitis. Intraoperative complications in terms of perforation of the sinus membrane were observed in 9.8% (*n* = 11) of the cases and postoperative complications in 15.1% (*n* = 17). Four out of 11 patients (36.3%) with membrane perforation experienced postoperative complications accompanied by swelling and wound infections. Local wound dehiscence without fluctuance was developed 10–14 days after surgery in 11 of these cases. In four cases, abscess was developed postoperatively. Symptoms of acute sinusitis, such as nasal congestion, headache, diffuse pain on the operated facial site, and fever or redness, were diagnosed in two patients 1 week after sinus floor elevation. According to their medical history, none of those patients had pathological findings in the sinus in the past. Despite the appropriate treatment and antibiotic therapy, the graft resorption was extremely providing no sufficient bone for implant placement. These two patients refused any additional surgical treatment. No postoperative hypoesthesia in the area of the infraorbital nerve was reported. A total of 166 dental implants were placed with satisfactory primary stability in the augmented areas; among them, 16 implants were placed in a simultaneous one-stage procedure in 16 sinus lifts in 16 patients after harvesting intraoral bone. The rest 150 implants were inserted in a secondarily two-stage procedure. None of the implants were lost during the healing period, and all implants showed osseointegration at the time of implant exposure.

### Nerve damage

**Fig. 4:**
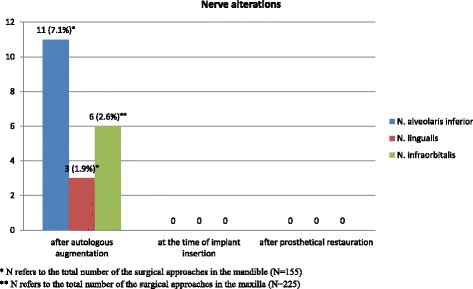
Postoperative nerve alterations. *Single asterisk*, *N* refers to the total number of the surgical approaches in the mandible (*N* = 155). *Double asterisk*, *N* refers to the total number of the surgical approaches in the maxilla (*N* = 225)

No permanent damage to any trigeminal nerves was evident in any of our entire cohort. All cases of postoperative hypoesthesia of the mental, lingual, or infraorbital nerve were just a temporary nature. At the time of implant surgery, none of these patients reported any persisting neural disturbances (Fig. 4).

In eleven patients, hypoesthesia of the mental area was mentioned, and three of them also reported sensation disturbance in the tongue after the harvesting and transplanting of a bone block from the mandibular ramus. However, in all of these cases of neural dysfunction, the recipient site for the grafts was in the mandible, so that, it was not possible to evaluate whether the nerve disturbances were caused by the harvesting of the bone block, by the augmentation procedure due to manipulation of the mental nerve or even by the inferior alveolar nerve block. None of the patients mentioned any isolated hypoesthesia in the lingual area. Infraorbital nerve hypoesthesia was reported postoperatively by two patients after the harvesting of grafts from the zygomatic buttress. There was no incidence of nerve disturbances after bone harvesting from the mandibular symphysis or from the iliac crest, or after sinus lift procedures.

### Perforation of the Schneiderian membrane

Sinus membrane perforation was observed intraoperatively in 11 of the 112 elevated sinuses (9.8%). After such perforations, postoperative complications accompanied by significant swelling and wound infection could be seen in four of these cases. Antibiotic treatment made the healing process absolutely effective, and dental implants could later be successfully inserted in all of these cases.

### Postoperative complications

Wound infections were observed in 2.6% of the donor sites (*n* = 8/300) and in 5.8% of the recipient sites (*n* = 22/378), while soft tissue dehiscences such as incision line opening occurred in 6.3% of the recipient sites (*n* = 24/378). Graft exposure was diagnosed in 21 of the 378 recipient sites (5.5%), while maxillary sinusitis occurred in 0.5% of the cases (*n* = 2/378), and only one patient (*n* = 1/378; 0.26%) suffered from postoperative hemorrhage. A detailed list of the postoperative complications that occurred after bone augmentation is given in Table 3.

### Complication management

Regarding intraoperative complications, all sinus membrane perforations were covered with a resorbable collagen membrane (Bio-Gide®, Geistlich Biomaterials, Baden-Baden, Germany) which applied as sealant to overlap the site of perforation prior to insertion of the graft material. These patients were advised to avoid physical stress, blowing their noses, or sneezing for a period of 3 weeks, and nose decongestant drops were prescribed.

Regarding postoperative swelling following the bone grafting procedure, most of the patients suffered a minimal facial deformity lasting not longer than 3–5 days. Swelling was so and otherwise an expected complication after surgery. At 2 weeks after the operation, none of the 179 patients reported persistent pain at the donor or recipient site.

Great importance was placed on the management of the postoperative complications. Minor effects such as soft tissue dehiscence with or without membrane exposure were treated conservatively with chlorhexidine mouth rinse (0.2%) and antibiotics either oral or intravenous (Augmentan^®^, GlaxoSmithKline Consumer Healthcare GmbH & Co. KG) achieving healing by secondary granulation. Patients with wound infection (*n* = ?) in the form of abscess formation had to be surgically drained, and systemic antibiotics were administered. In case of graft exposition without screw loosening (*n* = ?), the surgical field was revised, the bone block was refreshed with a diamond burr, and the flap was tensionlessly re-closed in combination parallel to antibiotic therapy. In case of graft exposure with screw mobility (*n* = ?), the bone grafts had been removed. All these patients were then scheduled to regular control appointments. Patients with symptoms of acute maxillary sinusitis after augmentation of the posterior maxilla (*n* = ?) were treated with antibiotic therapy and use of corticosteroid nasal spray for a period of 2 weeks.

### Implant placement

The overall implant success rate was 99.2%. All implants, without exception, were placed with guided surgery after implant planning using the coDiagnostiX^®^ software (Dental Wings GmbH, Chemnitz, Germany). Guided implant surgery was performed using insertion templates as described by Naziri et al. in all cases [34].

The average healing period until implant placement after bone harvesting was 4.53 months. Initially, 546 implants in 279 patients were planned. After the healing period, it was possible to place 525 implants in 436 successfully augmented areas in 259 patients. Three hundred implants were inserted in the maxilla and 225 in the mandible. The remaining 21 implants planned for 20 patients could not be placed. In six patients, an additional simultaneous augmentation with bone chips harvested with a bone scraper device was necessary to ensure that the entire implant shoulder was covered with bone. Two of these six patients had undergone augmentation with zygomatic buttress grafts, while two had received grafts from the mandibular ramus and two from the iliac crest.

A final rehabilitation with dental implants was possible in 97.9% of the patients (273 of 279). In the 14 patients who underwent re-augmentation due to primary graft failure, 15 implants could be successfully inserted later. The cases of implantation in a second time after re-augmentation were not included in our statistical evaluation. In the remaining six patients after graft failure, no re-augmentation was performed and no further implant treatment was desired.

Only two implants had to be removed from two patients before final prosthetic restoration due to lack of osseointegration, resulting in an early implant failure rate of 0.38%. These two implants were inserted in sites grafted with bone from the iliac crest. Both patients were smokers and had an SBI ≥ 20. The API of one of these two patients was also ≥20. With the exception of these two implants, all (523/525) were fully osseointegrated at the time of reentry for implant uncovery according to the Kerschbaum and Haastert criteria [35]. All of the implants were successful directly after prosthetic loading, based on the criteria of Buser et al. [34].

After prosthetic rehabilitation, all aspects of oral function were completely re-established in all patients. Two of 523 implants were lost within 2 years after prosthesis loading due to peri-implantitis. These data reveal a survival rate of 99.6% 2 years after prosthetic restoration.

**Fig. 5:**
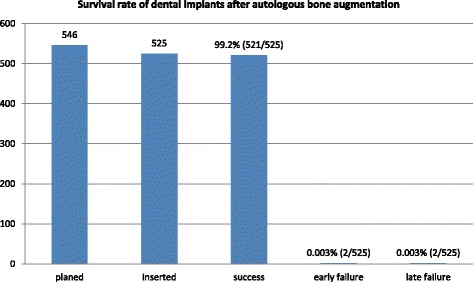
Survival rate of dental implants after autologous bone augmentation

The surgical outcome after augmentation and implantation procedures is presented in Fig. 5.

## Discussion

Several grafting procedures have been described to create sufficient volume of bone for implant placement [8, 9]. Autologous bone grafts can be harvested by an intraoral approach (mandibular ramus, mandibular symphysis, zygomatic buttress) or from distant sites (iliac crest, calvaria, and etc.) [17, 36, 37]. However, bone harvesting potentially causes donor site morbidity which is a major issue for the patients who appreciate procedures that reduce morbidity [38]. In order to decrease surgery duration and donor site discomfort using autologous bone sources, bone substitutes from various bone origins such as allografts and xenografts for use in reconstructive implant surgery had been developed. The postulation that bone alternatives could successfully substitute the use of autologous bone and its osteoinductive, osteoconductive, and osteogenic properties is under consideration, and various studies have proven the benefits and appropriateness of its material for an ideal reconstruction of some selected atrophic ridges prior to implantation [19, 24].

The advantages of the use of autologous bone harvested for alveolar reconstruction must be carefully evaluated, while most of the studies have focused mainly on the reconstructive procedure at the recipient site or on the complications related to harvesting, and only a very limited number of studies have reported the final results of the augmentation procedure [39–41].

The present study aimed to evaluate the clinical outcome of augmentation and implant success rate after autologous bone harvesting from different donor sites and the following possibility and adequacy of implant insertion in grafted areas and on the potential risk factors for postoperative discomfort and graft or implant failure. In our patient study group, we evaluated the survival rate of grafts harvested from the mandibular ramus (retromolar region), symphysial region, alveolar zygomatic buttress, the anterior and lateral facial wall (sinus floor elevations), and the anterior superior iliac crest as well as the implant success rate in a period of 2 years after prosthodontic restauration.

Systematic reviews have failed to find evidence that one particular grafting technique is superior to others [10]. Intraoral bone grafts from the mandibular symphysis, mandibular ramus, and maxillary tuberosity provide a good treatment modality for ridge augmentation, and the amount of bone available for harvesting is sufficient for defects up to the width of three teeth [42]. Harvesting of retromolar and symphysis bone grafts are especially recommended in cases involving multiple tooth reconstruction in the mandible. The access to the symphysis has been described as being easier than that to the mandibular ramus [43]. Both techniques can be performed on an outpatient basis, while harvesting of bone from distant sites is associated with inpatient care and increased costs [44]. It is reported that both harvesting procedures are well accepted by the patients, but the ramus was preferred [9, 41, 45]. Both the harvesting and grafting procedures are usually performed in the same surgical field.

In our study, seven out of 104 retromolar grafts and one out of 11 symphysis grafts have been lost due to postoperative complications; this is in accordance with the study review of Nkenke and Neukam [9]. They demonstrated also the low graft failure rate and the well acceptance of these methods by the patients. The ramus should be considered the site of choice when block grafts are needed for horizontal or vertical augmentation or for unilateral sinus augmentation [39]. For bilateral sinus elevation or when a combination of sinus elevation and horizontal and/or vertical augmentation are needed, the symphysis or iliac crest should be evaluated as a possible donor site [46]. When distant donor sites have to be adopted, it can be assumed that the morbidity and complication rate arising from the iliac crest is low [9]. This is in accordance to our study results; only one patient mentioned postoperative discomfort on the recipient site and only eight out of 116 bone grafts had to be removed due to infection.

Postoperative morbidity after mandibular bone harvesting procedures was reported to be mainly related to temporary or permanent neural disturbances involving the inferior alveolar nerve and its branches [19]. In this study, only the incidence of the temporary hypoesthesia of the mandibular and lingual nerve after harvesting from the retromolar area could be detected. It was 10.4 and 2.8%, respectively, results that are suitable to the literature [9, 47]. No consistent nerve damage could be detected. All of them made a complete recovery over the short and medium term. The use of piezoelectric surgery may reduce the incidence of this complication offering a safer way of safely removing hard tissue without damaging soft tissue. However, it should not be forgotten that piezoelectric surgery has some critical disadvantages, including a longer operation time and heat generation during bone cutting [48]. Based on our study results, however, we can recommend this technique as a safe method to prevent nerve damage in autologous intraoral bone harvesting. More histological studies are needed to define the quality of the retromolar bone and its resorption in the follow-up evaluation. That could minimize the early resorption rate and optimize the surgical outcome.

Harvesting from the zygomatic buttress is a relative new method [49]. This technique is best suited for those situations where only moderate amounts of bone are needed, especially when implant surgery is undertaken in the maxilla in one to two dental regions. The convex cross-section of the bone graft is ideal for the reconstruction of alveolar projection loss in the anterior and posterior maxillary zone. The zygomatic buttress is a strong bony pillar providing pressure absorption and transduction in the facial skeleton. With the described technique, it is possible to harvest approximately 0.5 to 1 ml of bone without causing damage to surrounding tissues. Low morbidity in donor and recipient sites are mentioned. This donor site offers easy access with excellent visibility and yields good quality bone of correct morphology and has the great advantage that no muscles have to be detached, and the bony structure in this area is especially strong [17, 49]. This clinical experience of fewer difficulties in managing postoperative edema and pain following this method was also presented from Gellrich et al. [49]. Limiting factors are the mucous membrane of the adjacent maxillary sinus and the close relationship to the infraorbital foramen [50]. Further studies to the bone quality of this potential donor site in order to minimize the postoperative resorption rate are necessary.

Of the sinus floor elevations performed in this study, 84.8% were defined absolutely successful. Only two of our 72 patients having sinus lift operations could not finally be treated with dental implants. These results are comparable to other studies considering the sinus graft to be a safe treatment modality with few complications [6, 8, 51–53]. Raghoebar et al. reported incidences of sinus complication of less than 1% in 100 patients [54]. Perforation of the sinus membrane has often been reported as the most common intraoperative complication in case studies with a frequency between 10 and 30% [53, 55, 56]. In this study, this was observed in 9.8% of the cases. On the other side, Scarano et al. demonstrated a high number of successfully treated patients with implant survival rate of 98.0% 4 years after augmentation using biomaterials [57]. Garofalo supports maxillary sinus elevations with biomaterials as a safe oral surgery technique with rapid and optimal bone regeneration leading to anatomical and functional restoration [58]. It is still under discussion if the use of autologous bone is superior to bone substitutes in sinus floor elevation procedures [59].

A previous systematic review reported 5% soft tissue complications after augmentation of fenestration- and dehiscence-type bone defects using resorbable membranes [60]. Soft tissue dehiscences or infections in the early postoperative phase preceded 6.3% of the cases in our study. The increased risk of bone block resorption in case of dehiscence is well described, which underlines the importance of meticulous soft tissue handling and tension-free soft tissue closure [61]. Postoperative infection of the donor and the recipient site preceded 2.6 and 5.6% of our cases, respectively, which is in accordance with complication rates reported in previous studies [62, 63]. Ponte and Khoury reported five cases of graft exposure in 521 treated patients, which results in a complication rate of less than 1% [64]. In our study, graft exposure occurred in 5.5% of the cases. Reviewing the literature, Jensen and Terheyden reported a complication rate of close to 18% [10]. In particular, if a bone block was grafted for ridge augmentation, the complication rate was higher, reaching 29.8% [10].

The use of autologous bone in this study has shown excellent graft survival and success rate (95.6%). This is equal to the results from the studies on implants inserted in reconstructed sites [6, 8, 24]. The early implant survival rate of 99.7% found in the present material is very high comparable to that in the previous systematic reviews after staged horizontal ridge augmentation [9, 10, 22, 62, 65]. The implant survival rate of 99.2% within 2 years after prosthodontic restauration in this study is higher compared to that in studies of the international literature with implants placed in autologous grafted areas [9]. Even with complete resorption of the grafted bone, an implant survival rate can be reached [9, 66]. However, this high rate of implant survival reported in our cases has to be confirmed due to further studies with a longer time period of control after prosthodontic rehabilitation. There is evidence that ridge augmentation success rates were 92 to 100% for onlay bone grafts and implant survival rates were 90.4% for onlay grafts [20–22]. In our study, the graft total success was determined by 95.6%, proving the high effectiveness of autologous bone harvesting even using extraoral donor sites.

One of the most serious problems associated with the use of bone blocks could be their resorption at the recipient site. The literature shows variations of this resorption from 25 to 60% [9, 67]. A systemic review reported additional augmentation in 26.6% of cases using a particulate augmentation protocol and in 4.7% of the bone block cases [10]. Widmark et al. reported bone resorption after lateral bone block augmentation of up to 60% of the original bone graft volume at the time of implant abutment connection [68]. No radiological evaluation of the quantity and quality of the autologous bone harvested from different donor sites was carried out in this study. A lower percentage of 1.3% (6/436) of additional augmentation procedures at the time of implant placement was found in the present study and was not considered as primary complication [69]. The authors attributed the low graft resorption to the short healing period of 3 to 5 months, which has now proven to be sufficient for the revascularization of the graft and the secure insertion of dental implants as suggested in the past by various authors [70, 71]. Therefore, we suggest limiting the healing period after autologous bone grafting to a maximum of 3 to 6 months. This leads to earlier dental restorations compared to augmentations using bone substitutes, where the healing periods are often recommended up to 12 months. Strategies to minimize bone resorption after autologous grafting are discussed in the literature. However, further studies are necessary to identify factors influencing bone resorption [47, 72].

The results of the present study have to take into account the absence of a control group with patients undergoing bone augmentation procedures with bone substitutes (allogen, alloplastic, exogen). Without a comparative group of grafting surgeries using alternative bone material, only limited statements can be made.

However, the excellent surgical outcome of autologous surgical methods providing high survival rate of the grafts and inserted implants in our study proves for the first time the reliability and low comorbidity of autologous bone grafts in preprosthetic surgery for almost all kinds of intraoral and even for extraoral grafts from the iliac crest. Despite the study had a retrospective design, and the nature of a retrospective study inherently results in flaws, in this study there was no dropout of any patient operated in the years 2009–2011, and all patients operated with bone grafts prior to implant insertion in our department had been included in the study. This moreover reflects a prospective study design, which has never been reported in the literature before.

No evidence in the literature comparing surgical outcomes of autologous augmentations including postoperative morbidity from different donor sites, subject to graft and implant survival, has been reported. The relative short-term period of implant control after the prosthetic rehabilitation could be critical in order to define the implant success rate in augmented sites. Related to that, there was also no control group with implants placed in non-grafted areas that could facilitate the comparison of augmented and non-augmented areas. There was also no radiological evaluation of the graft resorption after harvesting; our findings were after clinical observation that could jeopardize the real dimensions of the graft at the time of implantation because of the missing comparison between pre- and post-augmentative situations. Future research could include control groups with large cohort size, long-term follow-up periods, and standardized criteria for defining bone graft and implant success or failure rate, in order to obtain rigorous evidence-based results. This will most likely only be achievable as single-arm multicenter studies.

Data on risk factors based on the original examination and documentation are difficult to assess the adverse effects of variable factors on the surgical prognosis because of the multifactorial genesis of surgical complications [73]. Factors such as gender, age, or smoking habit could be associated with postoperative complications after two-stage dentoalveolar reconstruction with autologous bone grafts. For example, a higher ratio of men compared to women (250 men to 29 women) was detected in this study. This can be logically explained due to the profession of the patients. The study was carried out in the military hospital of Ulm in Germany and the majority of the collective concerned male candidates. However, this inhomogeneity could significantly influence on the postoperative surgical outcome. It is a fact that conditions of the female nature, such as estrogen level alteration in postmenopausal women, may relate to bone graft resorption. To investigate the age-specific impact on the prognosis of these procedures, a prospective case control study in equal age and gender groups of patients is required. Possible risk factors for postoperative morbidity regarding complications at the donor and recipient sites after augmentation procedures with autologous bone grafts harvested from different donor sites were not analyzed in this study. An attempt to estimate the frequency with which postoperative complications occur in patients with different characteristics, such as age, gender, smoking habit, history of periodontitis, and more, in order to evaluate the influence of these factors on augmentation outcome and implant survival rate should be aimed in future studies.

Therefore, it has to be kept in mind that equal alternatives to autologous bone grafts in sinus floor augmentation are proven. It seems that the use of bone substitutes finally leads to implant survival rates that are comparable to those that can be achieved with implants placed in sinuses grafted with autologous bone [74]. In this respect, assessment of the defect should be carefully performed in order to provide the patient with the least invasive technique providing excellent long-term results. Our technique of using autologous bone scrapes from the anterior facial wall harvested during window preparation has proven to be equal to the use of bone substitutes. This technique combines augmentation with autologous material and the absence of comorbidity at the donor site. Surgeons therefore should consider using this technique in order to minimize the use of bone substitutes without additional harvesting morbidity.

## Conclusions

The results of the clinical study proves the reliability and low comorbidity of autologous bone grafts in preprosthetic alveolar ridge reconstructions prior to implant insertion. The high graft success rate (95.6%) and the low early implant failure rate (0.38%) in a surveillance of all patients treated in three following years with this technique showing no exclusion and no dropout of any case for the first time proves that intraoral and extraoral autologous bone grafts could be further considered as the “gold standard” preprosthetic dentoalveolar reconstruction. This study demonstrates that the reconstruction of atrophic jaws with corticocancellous bone grafts from intraoral and extraoral donor sites is a predictable technique to facilitate dental rehabilitation of the atrophic ridge, associated with high bone survival rate and implant success. Although an increased number of bone substitutes exist, autologous bone has to be considered as the most effective material for two-stage pre-prosthodontic augmentation in oral implantation. Intraoral autologous grafts can serve as a reliable treatment option to reconstruct isolated defects for implant placement. Autologous onlay grafts from the ramus mandible, symphysis, and zygomatic buttress offer sufficient bone volume to reconstruct the atrophic jaw without any lasting harvesting morbidities. These sites are excellent treatment alternatives with high patient acceptance when reconstruction is necessary before implant insertion. Besides a successful reconstruction of the alveolar crest with correct selection of the donor site, patient acceptance of the procedure should be high, while the morbidity of the procedure should be minimal showing no persistent nerve damage, which was considered the main disadvantage of autologous bone grafts in the past. We cannot address the contribution of the use of piezoelectric surgery, grafting templates, and guided implant insertion to the reported results, but we could proof that with the described technique, predictable outcome lacking lasting morbidities can be achieved independently from surgical status such as residents, fellows, or consultants. Further studies should focus on long-term implant success rates in these patients. The influence of different patient characteristics as potential risk factors for postoperative morbidity may also be of interest for further investigations.
